# The *CRKL* gene encoding an adaptor protein is amplified, overexpressed, and a possible therapeutic target in gastric cancer

**DOI:** 10.1186/1479-5876-10-97

**Published:** 2012-07-03

**Authors:** Hiroko Natsume, Kazuya Shinmura, Hong Tao, Hisaki Igarashi, Masaya Suzuki, Kiyoko Nagura, Masanori Goto, Hidetaka Yamada, Matsuyoshi Maeda, Hiroyuki Konno, Satoki Nakamura, Haruhiko Sugimura

**Affiliations:** 1 Department of Tumor Pathology, Hamamatsu University School of Medicine, 1-20-1 Handayama, Higashi Ward, Hamamatsu, Shizuoka, 431-3192, Japan; 2 Department of Pathology, Toyohashi Municipal Hospital, 50 Hachiken Nishi, Aotake-cho, Toyohashi, Aichi, 441-8570, Japan; 3 Second Department of Surgery, Hamamatsu University School of Medicine, 1-20-1 Handayama, Higashi Ward, Hamamatsu, Shizuoka, 431-3192, Japan; 4 Third Department of Internal Medicine, Hamamatsu University School of Medicine, 1-20-1 Handayama, Higashi Ward, Hamamatsu, Shizuoka, 431-3192, Japan

**Keywords:** CRKL, Gastric cancer, Cell proliferation, Overexpression, Copy number amplification

## Abstract

**Background:**

Genomic DNA amplification is a genetic factor involved in cancer, and some oncogenes, such as *ERBB2*, are highly amplified in gastric cancer. We searched for the possible amplification of other genes in gastric cancer.

**Methods and Results:**

A genome-wide single nucleotide polymorphism microarray analysis was performed using three cell lines of differentiated gastric cancers, and 22 genes (including *ERBB2*) in five highly amplified chromosome regions (with a copy number of more than 6) were identified. Particular attention was paid to the *CRKL* gene, the product of which is an adaptor protein containing Src homology 2 and 3 (SH2/SH3) domains. An extremely high *CRKL* copy number was confirmed in the MKN74 gastric cancer cell line using fluorescence *in situ* hybridization (FISH), and a high level of CRKL expression was also observed in the cells. The RNA-interference-mediated knockdown of CRKL in MKN74 disclosed the ability of CRKL to upregulate gastric cell proliferation. An immunohistochemical analysis revealed that CRKL protein was overexpressed in 24.4% (88/360) of the primary gastric cancers that were analyzed. The *CRKL* copy number was also examined in 360 primary gastric cancers using a FISH analysis, and *CRKL* amplification was found to be associated with CRKL overexpression. Finally, we showed that MKN74 cells with *CRKL* amplification were responsive to the dual Src/BCR-ABL kinase inhibitor BMS354825, likely via the inhibition of CRKL phosphorylation, and that the proliferation of MKN74 cells was suppressed by treatment with a CRKL-targeting peptide.

**Conclusion:**

These results suggested that CRKL protein is overexpressed in a subset of gastric cancers and is associated with *CRKL* amplification in gastric cancer. Furthermore, our results suggested that CRKL protein has the ability to regulate gastric cell proliferation and has the potential to serve as a molecular therapy target for gastric cancer.

## Background

Although the overall incidence of gastric cancer is decreasing in many countries, the high incidence of gastric cancer remains a serious health problem, and gastric cancer continues to be the second-leading cause of cancer-related death worldwide [1,2]. Gastric carcinogenesis is a multi-step process in which environmental and genetic factors interact [1-8]. Among the genetic changes observed in cancerous cells, genomic DNA amplification is a well-known alteration that is involved in gastric cancer [4,5,7]. Amplification is often associated with increased expression levels of the genes contained in the amplified loci [5]. Oncogenes in gastric cancer, such as *MYC* (mapped to chromosome 8q24), *KRAS* (12p12), and *ERBB2* (17q12), are located in such amplified regions [4,5,7,9]. We considered the possibility that there exist genes whose amplification in gastric cancer has not been revealed to date. To uncover such novel gene alterations, we searched for highly amplified genes in gastric cancer using a genome-wide single nucleotide polymorphism (SNP) microarray analysis and found that the *CRKLv-crk sarcoma virus CT10 oncogene homolog (avian)-like* gene (22q11) is highly amplified in gastric cancer. The CRKL, a member of the CRK family of adapter proteins, consists of an NH2-terminal Src homology 2 (SH2) domain followed by two SH3 domains: SH3n and SH3c [10], and participates in signal transduction in response to growth factors, cytokines, and the oncogenic BCR-ABL fusion protein, resulting in cell proliferation, survival, adhesion, and migration [10,11]. We hypothesized that CRKL might play an important role in gastric carcinogenesis and investigated whether CRKL expression and the function of CRKL protein affect the regulation of cell proliferation in gastric cancer. We also investigated responsiveness of a gastric cancer cell line containing *CRKL* amplification to a kinase inhibitor, BMS354825, and a CRKL-targeting peptide.

## Materials and Methods

### Cell lines and surgical specimens

The gastric adenocarcinoma cell lines MKN7, MKN28, MKN74, and AGS were purchased from the Human Science Research Resource Bank (Osaka, Japan) or from American Type Culture Collection (Manassas, VA). Cells were cultured and grown in RPMI 1640 medium supplemented with 10% fetal bovine serum, penicillin (100 units/mL), and streptomycin (100 μg/mL) under a 5% CO_2_ atmosphere at 37°C. Paraffin-embedded gastric tissues obtained from gastric cancer patients who underwent surgery at Toyohashi Municipal Hospital (Japan) were used for the immunohistochemical analysis. Gastric tissue samples obtained from gastric cancer patients who underwent surgery at Hamamatsu University Hospital (Japan) were used for the quantitative reverse-transcription (QRT)-polymerase chain reaction (PCR) analysis. The study design was approved by the Institutional Review Boards (IRBs).

### Genome-wide SNP microarray

**Figure 1:**
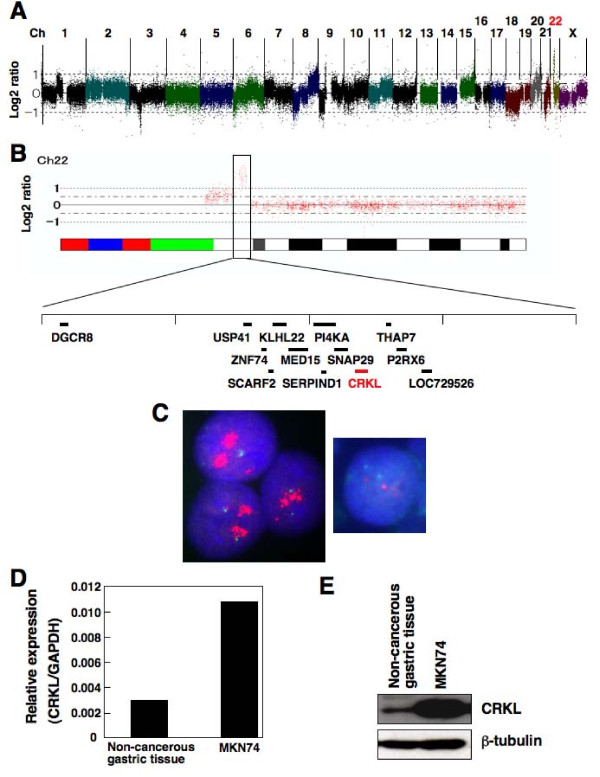
**Identification of highly amplified chromosome regions containing the*CRKL*gene and the detection of CRKL overexpression in gastric cancer. (A)** Genome-wide detection of copy number alterations using a high-density SNP microarray in the MKN74 gastric cancer cell line. The copy number status for the whole genome of MKN74 is shown. DNA (250 ng) was analyzed using an Affymetrix GeneChip 250 K NspI array, and the total copy numbers were determined by analyzing the microarray data using the CNAG program. The chromosome number is shown above the panel. Chromosome 22 is highlighted in red. **(B)** The copy number status of chromosome 22 of the MKN74 cells is shown. A highly amplified region of chromosome 22 is enlarged, and the genes located in this region are indicated. The *CRKL* gene is highlighted in red. **(C)** Detection of *CRKL* amplification in MKN74 cells using a FISH analysis. The left panel shows the *CRKL* signal (red) in MKN74 cells, while the right panel shows the *CRKL* (red) in non-cancerous gastric tissue cells. An extreme increase in the *CRKL* copy number was observed in the MKN74 cells, while a normal copy number (2) was seen in non-cancerous cells. Nuclei are stained with DAPI. **(D)** Detection of the increased expression of CRKL mRNA transcript in MKN74 cells using real-time QRT-PCR analysis. The amounts of CRKL transcripts normalized to the amount of GAPDH transcripts are shown in the graph. The average expression level of eight normal gastric mucosa samples was measured as a control. **(E)** Detection of the increased expression of CRKL protein in MKN74 cells using a western blot analysis. The expression of CRKL was examined using anti-CRKL monoclonal antibody (Y244; 1:500 dilution), horseradish peroxidase-coupled secondary antibody (1:5,000 dilution), and enhanced chemiluminescence detection reagents. The expression of β-tubulin protein was analyzed as an internal control.

DNA (250 ng) was digested with *Nsp*I restriction enzyme (New England Biolabs, Hertfordshire, UK) and ligated to a universal adaptor sequence. The ligated DNA was PCR-amplified using primers complementary to the universal adaptors, and the PCR products were purified, quantified, and normalized. The products were then fragmented, end-labeled using terminal deoxynucleotidyl transferase, and hybridized to the Affymetrix GeneChip human mapping 250 K NspI arrays (Affymetrix Japan, Tokyo, Japan). After hybridization, the arrays were washed, stained using Affymetrix fluidics station 450, and scanned with a GeneChip Scanner 3000 7 G. Raw SNP call data were extracted using Affymetrix GeneChip Genotyping Analysis software (GTYPE) 4.1. The SNP microarray data were analyzed to determine the total copy number using the CNAG program, as previously described [12,13] (Figure 1).

### WST-8 assay

**Figure 2:**
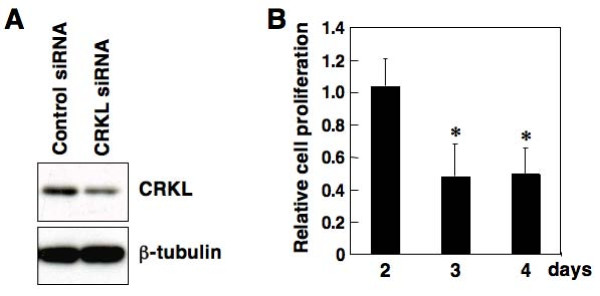
**Ability of CRKL to regulate cell proliferation in the MKN74 gastric cancer cell line. (A)** siRNA knockdown for CRKL in MKN74 cells with *CRKL* amplification. Cells were reverse-transfected with the siRNA oligonucleotides (20 nM) using HiPerFect Transfection Reagent, and 8 × 10^5^ cells were seeded in 60 mm-dishes with 4 ml media. The expression of CRKL was examined 4 days after the reverse transfection of CRKL siRNA or negative control siRNA using a western blot analysis with anti-CRKL monoclonal antibody (Y244; 1:500 dilution). The level of CRKL protein expression was decreased in CRKL siRNA-transfected cells, compared with mock-transfected cells. The expression of β-tubulin protein was analyzed as an internal control. **(B)** Decrease in the proliferation of MKN74 cells transfected with siRNA for CRKL. Cells were reverse-transfected with the siRNA oligonucleotides (20 nM), and 1.5 × 10^4^ cells were seeded in 96-well microplates containing 100 μL of media. After the reverse transfection of CRKL siRNA or negative control siRNA, the number of viable cells was counted by measuring the reduction in the tetrazolium monosodium salt WST-8. The cell number of CRKL siRNA-transfected cells relative to that of mock-transfected cells is shown. Values are the mean ± standard deviation of three independent experiments. *P*-values were calculated using the *t*-test, and * indicates a statistical significance.

Cell proliferation and viability were quantified using a Cell Counting Kit-8 (Dojindo, Kumamoto, Japan) according to the manufacturer’s instructions [14]. The assay was based on the extracellular reduction of the tetrazolium salt WST-8 by NADH produced in the mitochondria of living cells. The cells were incubated with the WST-8 reagent for 1 hr at 37°C, and the absorbance was measured at 450 nm using an EL340I microplate reader (BIO-TEK Instruments, Winooski, VT) (Figure 2).

### Immunohistochemistry

**Figure 3:**
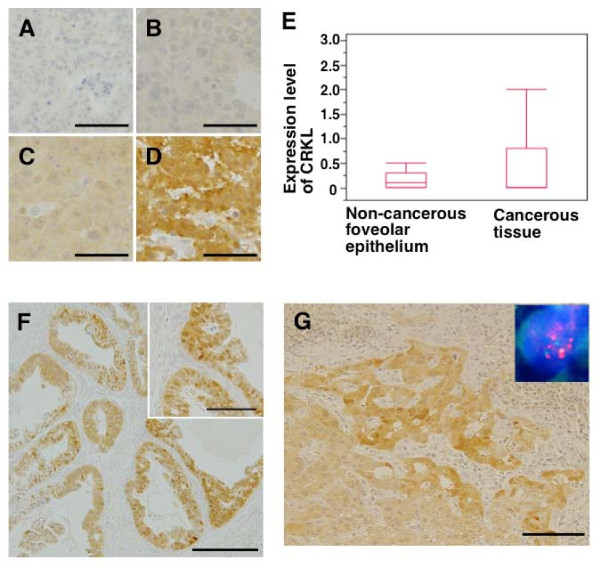
**Immunohistochemical detection of CRKL protein in primary gastric cancer.** TMA block sections were subjected to an immunohistochemical analysis using anti-CRKL monoclonal antibody (Y243; 1:100 dilution), Histofine Simple Stain Max-Po (Multi), and 3,3'-diaminobenzidine tetrahydrochloride. Intensity values of 0, 1, 2, and 3 are shown in **(A)**, **(B)**, **(C)**, and **(D)**, respectively. Bar = 50 μm. **(E)** Box-plot analysis of CRKL protein expression in gastric tissue. A statistically significant difference in the CRKL expression level was detected between non-cancerous gastric foveolar epithelium (*n* = 41) and gastric cancerous tissue (*n* = 360). **(F)** Representative result of the CRKL immunohistochemical analysis. A gastric cancer with a high CRKL expression level is shown. Bar = 500 μm. The inset is a magnified image. Bar = 50 μm. **(G)** Representative gastric cancer case showing both a high CRKL expression level and *CRKL* gene amplification. The high CRKL expression level (value = 2.6) was detected using an immunohistochemical analysis. Bar = 100 μm. The inset shows the amplification of *CRKL* (red) in the cancer cells. The *CRKL* signal (red) and the control signal for chromosome 22 (green) were detected using a FISH analysis. Nuclei are stained with DAPI.

Tissue microarray (TMA) blocks were prepared as previously described [14-16]. TMA block sections were deparaffinized, rehydrated, and boiled in Tris-EDTA buffer (pH 9.0) for antigen retrieval. Endogenous peroxidase activity was blocked by incubation in a hydrogen peroxide solution. Next, the sections were incubated with a rabbit anti-CRKL monoclonal antibody (Y243; Abcam, Cambridge, UK). The antigen-antibody complex was visualized using Histofine Simple Stain Max-Po (Multi) (Nichirei, Tokyo, Japan) and 3,3'-diaminobenzidine tetrahydrochloride. Counterstaining was performed using hematoxylin. The intensity values of the cells were determined using a 4-point scale according to the color of the cell cytoplasm after CRKL immunostaining as follows: 0, blue; 1, blue-brown; 2, light brown; and 3, brown. The percentage of cells with each intensity value was then multiplied by the intensity value, as described previously [14]. The scores obtained for CRKL immunostaining were classified as either a low expression level (0–0.99) or a high expression level (1.00–3.00) (Figure 3) .

### DNA fluorescence *in situ* hybridization (FISH)

FISH was performed as previously described [16-19]. Tissue slides were hybridized with a Spectrum Orange-labeled BAC clone (RP11-801O20 and RP11-1058B20) for the *CRKL* locus (Advanced GenoTechs Co., Tsukuba, Japan) and a Spectrum Green-labeled control probe for the near centromere locus on chromosome 22 (BAC clone: RP11-232E17). 4',6-Diamidino-2-phenylindole (DAPI) (Vector Laboratories, Burlingame, CA) was used for nuclear staining (Figure 3).

### MTT assay and direct cell counting

**Figure 4:**
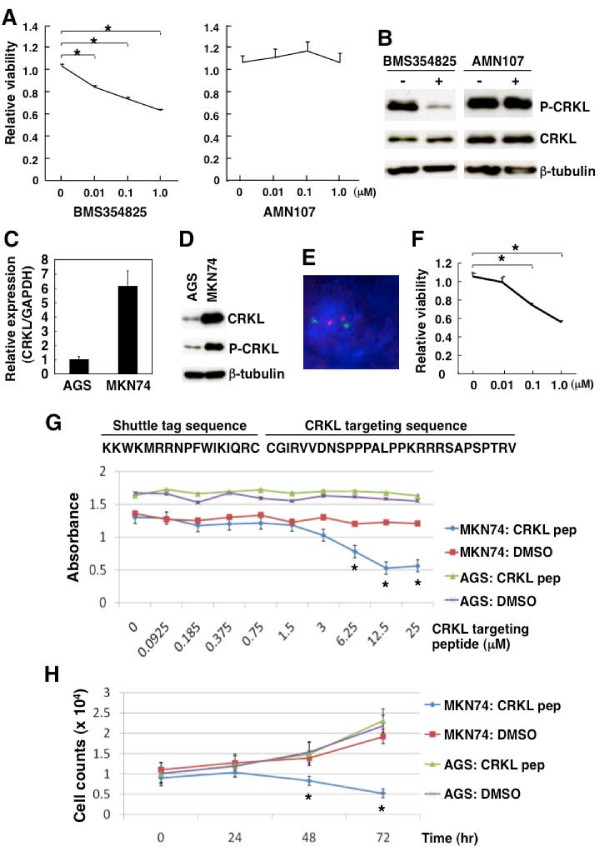
**Responses of the MKN74 gastric cancer cell line with*CRKL*amplification to treatment with BMS354825 (a dual Src/BCR-ABL kinase inhibitor) and CRKL-targeting peptide. (A)** Viability of MKN74 cells treated with BMS354825 but not those treated with AMN107 (a highly selective BCR-ABL kinase inhibitor) is decreased. The cells were seeded in 96-well microplates at a density of 1 × 10^4^ per well; after 24 h, the drug (0.01–1.0 μM) or 0.1% DMSO solution was added. Viability was examined in the MKN74 cells after 72 h of treatment at the indicated concentration using WST-8 reagent. The number of viable cells after treatment with each inhibitor was normalized to the number of viable cells without treatment, and the relative viability is shown in the graph. Values are the mean ± standard error. *P* values were calculated using the Dunnett’s multiple comparison test, and * indicates a statistically significant decrease. **(B)** Effective inhibition of CRKL phosphorylation in MKN74 cells treated with BMS354825. Cells were treated with each inhibitor (DMSO only or 0.01 μM of drug) for 90 min, and the expression of CRKL protein was examined using a western blot analysis with anti-phospho CRKL polyclonal antibody (Y207; 1:1,000 dilution) or anti-CRKL monoclonal antibody (Y244; 1:500 dilution). The expression of β-tubulin protein was analyzed as an internal control. **(C)** Comparison of CRKL mRNA transcripts between AGS and MKN74 cells using real-time QRT-PCR analysis. The amounts of CRKL transcripts normalized to the amount of GAPDH transcripts are shown in the graph. **(D)** Comparison of expression of CRKL protein between AGS and MKN74 cells using a western blot analysis. The expression of CRKL was examined using the primary antibodies shown in (B). The expression of β-tubulin protein was analyzed as an internal control. **(E)** Detection of *CRKL* gene copy number in AGS cells using a FISH analysis. The *CRKL* signal is red, and the control signal for chromosome 22 is green. Nuclei are stained with DAPI. **(F)** Viability of AGS cells decreased after BMS354825 treatment. Viability was examined as described in (A). Values are the mean ± standard error. *P* values were calculated using a *t*-test, and * indicates a statistically significant decrease. **(G)** MKN74 cells with *CRKL* amplification and AGS cells without *CRKL* amplification were seeded in 96-well microplates at a density of 1 × 10^4^ per well. 24 h after seeding, cells were treated with CRKL-targeting peptide (0.0925–25 μM) or 0.2% DMSO solution at the indicated concentration. The sequence of the CRKL-targeting peptide that was used is shown above the graph. After 72 h of incubation, viability was determined using an MTT assay. The results are presented as the mean ± standard deviation of three independent experiments. *P* values were calculated using a *t*-test, and * indicates a statistically significant difference between the cells treated with CRKL-targeting peptide and those treated with DMSO. **(H)** Cell proliferation of MKN74 and AGS cells treated with CRKL-targeting peptide (6.25 μM) or DMSO as measured by counting cells using a hemocytometer. Cells (1 × 10^4^) were seeded in 24-well plates and treated with CRKL-targeting peptide or DMSO. The cell counting was performed every 24 h for 3 days. Data are shown as the mean ± standard deviation of three independent experiments. *P* values were calculated using a *t*-test, and * indicates a statistically significant difference between the cells treated with CRKL-targeting peptide and those treated with DMSO.

In the experiment involving treatment with the CRKL-targeting peptide, an MTT assay was performed to assess cell viability in Figure 4G. The cells were cultured with the indicated concentration of CRKL-targeting peptide or dimethyl sulfoxide (DMSO) at 37°C for 72 h, and 3-(4,5-dimethylthiazol-2-yl)-2,5-diphenyltetrazolium bromide (MTT) solution (Sigma-Aldrich, St. Louis, MO) was then added at a final concentration of 0.25 mg/mL. After incubation at 37°C for 4 h, absorbance was measured at a wavelength of 570 nm using a microplate reader. Cells grown in complete medium with DMSO alone were used as controls. The final concentration of DMSO was set to 0.2%. To assess cell proliferation in Figure 4H, the cells were cultured with CRKL targeting peptide or DMSO at 37°C for 72 h. Cell proliferation was measured by directly counting the cells using a hemocytometer, as described previously [20].

### QRT-PCR

Total RNA was extracted using Isogen (Nippongene, Tokyo, Japan) or an RNeasy Plus mini kit (Qiagen, Valencia, CA) and converted to cDNA using the SuperScript First-Strand Synthesis System (Invitrogen, Carlsbad, CA). Real-time QRT-PCR was performed using the cDNA and Fast SYBR Green Master Mix (Applied Biosystems, Foster City, CA) on a StepOne Real-Time PCR system (Applied Biosystems). The following PCR primers were used: 5'-CAA CCT GCC TAC AGC AGA AGA TAA-3' and 5'-CGG CAT CAT TCC CAG GAA-3' for the CRKL transcript, and 5'-GGT GGT CTC CTC TGA CTT CAA CA-3' and 5'-GTT GCT GTA GCC AAA TTC GTT GT-3' for the transcript of a housekeeping gene, *GAPDH*. The relative amounts of CRKL transcript were normalized to those of the GAPDH transcript.

### Western blot analysis

Cells were lysed, and the protein concentration was quantified using a BCA protein assay kit (Pierce, Rockford, IL). The proteins were electrophoresed and transferred to a PVDF membrane (GE Healthcare Bio Science, Piscataway, NJ). After blocking with non-fat milk or Blocking One-P (Nakalai Tesque, Kyoto, Japan), the membrane was incubated with rabbit anti-CRKL monoclonal antibody (Y244; Abcam), rabbit anti-phospho CRKL polyclonal antibody (Y207; Cell Signaling, Beverly, MA), or mouse anti-β-tubulin (2-28-33, Sigma-Aldrich). The immunoreactive proteins were visualized using horseradish peroxidase-coupled secondary antibody and enhanced chemiluminescence detection reagents (GE Healthcare Bio Science) [21].

### Small interfering RNA (siRNA) knockdown

A stealth siRNA duplex oligonucleotide (Invitrogen) was used for siRNA knockdown. The following CRKL sequence was used: 5'-UCG UGA AAG UCA CAA GGA UGA AUA U-3'. A low GC Duplex #2 (Invitrogen) was used as a negative control. MKN74 cells were reverse-transfected with the siRNA oligonucleotides (20 nM) using HiPerFect Transfection Reagent (Qiagen), according to the manufacturer’s instructions.

### BMS354825 and AMN107 treatment

BMS354825, a dual Src/BCR-ABL kinase inhibitor, was kindly provided by Bristol-Myers Squibb (New York, NY), and AMN107, a highly selective BCR-ABL kinase inhibitor, was kindly provided by Novartis Pharmaceuticals (Basel, Switzerland) [22-25]. Stock solutions (10 mM) of BMS354825 and AMN107 were prepared in DMSO. The cells were incubated with BMS354825 or AMN107 at a final concentration of 0.01 to 1.0 μM for 72 h. The final concentration of DMSO was set to 0.1%.

### Preparation of CRKL targeting peptide

In this study, we used the peptides, which has been reported to be disrupted complexes between BCR-ABL and CRKL depend on the SH3 domain of CRKL in CML cells [26]. Peptides used in the experiments are followed: CRKL-targeting peptide; KKW KMR RNP FWI KIQ RC – CGI RVV DNS PPP ALP PKR RRS APS PTR V, control peptide; KKW KMR RNP FWI KIQ RC – CGI RVV DNS PPG ALG PLL RRS APS PTR V. The KKW KMR RNP FWI KIQ RC was the shuttle tag sequence performing a receptor-independent cell entry. The chimeric peptide was synthesized and purified by using reverse-phase high performance liquid chromatography (HPLC) (Toray Research Center, Otsu, Japan). Peptide stocks were prepared in DMSO and stored in aliquots at -80°C.

### Statistical analysis

The statistical analysis was performed using an unpaired *t*-test, chi-square test, or Dunnett’s test. JMP version 7.0.1 software (SAS Institute, Cary, NC) was used for the analyses. *P* values less than 0.05 were considered statistically significant.

## Results

### Identification of *CRKL* amplification in gastric cancer

To search for highly amplified genes in gastric adenocarcinoma, we adopted a genome-wide high-resolution SNP microarray approach in three cell lines of differentiated gastric adenocarcinoma: MKN7, MKN28, and MKN74. Genotype calls were obtained at more than 95% of the 262,264 SNP sites on the array, meaning that the SNP microarray analysis had been performed properly. The SNP microarray data were then used to determine the chromosomal copy number using the CNAG program (Figures 1A and 1B). Five highly amplified regions with a copy number of more than 6 (9p13, 17q12-q21, 19q12, 19q13, and 22q11) were identified, as shown in Table 1. These regions contained various kinds of genes, a total of 22 genes (Table 1). Among them, we decided to focus on the *CRKL* gene at chromosome 22q11.21, the product of which is an SH2 and SH3 domain-containing adaptor protein that shares homology with the CRK oncoprotein, because CRKL is a known substrate of BCR-ABL kinase in Philadelphia chromosome-positive leukemia [27,28] and its role in gastric cancer has not been previously analyzed. To confirm that *CRKL* gene amplification was detectable in the MKN74 cell line, we performed a FISH analysis using a probe specific for *CRKL*. As expected, an extreme increase in the *CRKL* copy number was detected in the MKN74 cells using a FISH analysis (Figure 1C). When the level of CRKL mRNA expression was examined in MKN74 cells using a real-time QRT-PCR analysis, the level was much higher than that in non-cancerous gastric tissue (Figure 1D). Moreover, a western blot analysis showed that the level of CRKL protein expression was higher in MKN74 cells than in non-cancerous gastric tissue (Figure 1E). These results suggested that the *CRKL* gene is highly amplified and that CRKL is overexpressed in a subset of gastric cancer cell lines.

**Table 1:** Detection of chromosomal regions with a high copy number (more than 6) in the gastric cancer cell lines MKN7, MKN28, and MKN74 using a genome-wide SNP microarray analysis

Chromosomal regions^a^	Genes with a high copy number in the region
9p13	*PAX5*
17q12-q21	*FBXL20, MED1, PERLD1, ERBB2, IKZF3, ZPBP2*
19q12	*CCNE1*
19q13	*CD22*
22q11	*DGCR8, USP41, ZNF74, SCARF2, KLHL22, MED15, PI4KA, SERPIND1, SNAP29, CRKL, THAP7, P2RX6, LOC729526*

^a^ If more than four consecutive SNP probes with a copy number of more than six were detected in either of the three cell lines, the chromosomal region was regarded as being a “highly amplified region” and was listed in this table.

### Ability of CRKL to control gastric cell proliferation

To explore the functional significance of *CRKL* amplification in gastric cancer, we attempted to examine the effect of overexpressed CRKL on gastric cell proliferation. For this purpose, we prepared MKN74 cells with distinct CRKL expression levels using the siRNA knockdown of CRKL expression. CRKL-specific siRNA transfection effectively decreased the level of CRKL protein expression in MKN74 cells by approximately 70% of the levels observed in negative control siRNA-transfected cells (Figure 2A). A cell proliferation assay showed that the number of CRKL siRNA-transfected MKN74 cells was significantly lower at 3 and 4 days after transfection than the number of negative control siRNA-transfected cells (Figure 2B), meaning that CRKL has the ability to upregulate cell proliferation.

### Overexpression of CRKL protein in gastric cancer

Next, we investigated the expression status of CRKL protein in primary gastric cancer using an immunohistochemical analysis with anti-CRKL monoclonal antibody (Y243). CRKL was mainly observed in the cytoplasm, consistent with previous reports [29]. When we compared the level of CRKL expression between non-cancerous gastric foveolar epithelium (*n* = 41) and gastric cancer (*n* = 360), the level of CRKL expression in gastric cancer (mean ± standard deviation = 0.42 ± 0.63) was significantly higher than that in non-cancerous tissue (0.20 ± 0.26) (*P* = 0.032) (Figures 3A3E). When an expression level of 1.00, which corresponds to a value 5-fold of the mean expression level in non-cancerous gastric foveolar epithelium, was used as a cutoff value for the expression status in gastric cancer (i.e., low expression group, 0–0.99; high expression group, 1.00–3.00), 88 (24.4%) of the 360 primary gastric cancers were included in the high expression group (Figure 3F). To examine whether CRKL overexpression is associated with *CRKL* amplification in gastric cancer, we performed a FISH analysis for the *CRKL* gene in the 360 primary gastric cancers and compared the prevalence of *CRKL* amplification between the low expression group and the high expression group. As expected, the percentage of gastric cancer cells with *CRKL* amplification was significantly higher in the high expression group (9.1%; 8/88 cases) than in the low expression group (2.2%; 6/272 cases) (*P* = 0.028, chi-square test). This result suggests that *CRKL* amplification contributes to CRKL overexpression in primary gastric cancer. We further investigated whether the levels of CRKL expression is associated with clinicopathological features in primary gastric cancer patients, the high CRKL expression was observed significantly more often in male and differentiated-type gastric cancer (Table 2). These results suggested that CRKL protein is overexpressed partly due to *CRKL* amplification in a subset of primary gastric cancers and is associated with the gender and histopathology.

**Table 2:** Association between CRKL expression and clinicopathological factors in 360 patients with primary gastric cancer

		CRKL expression level		
Factor	Patient	Low (*n* = 272)	High (*n* = 88)	*P*
Age				
Year, mean ± SD^a^	62.0 ± 11.2	61.7 ± 11.7	62.9 ± 11.4	0.3936^b^
(range)	(29–86)	(29–86)	(31–85)	
Gender				
Male	255	182 (66.9%)	73 (83.0%)	0.0028^c^
Female	105	90 (33.1%)	15 (17.0%)	
Histological type				
Differentiated	172	118 (43.4%)	54 (61.4%)	0.0033^c^
Undifferentiated	188	154 (56.6%)	34 (38.6%)	
pT stage				
pT1	143	103 (37.9%)	40 (45.5%)	0.2082^c^
pT2-pT4	217	169 (62.1%)	48 (54.5%)	

^a^ SD, standard deviation. ^b^*t*-test. ^c^ Chi-square test.

### Decrease in the viability of CRKL-expressing MKN74 cells treated with BMS354825

Finally, we tested the possibility of using CRKL as a therapeutic target in MKN74 cells with *CRKL* amplification. Since Philadelphia chromosome-positive leukemia expressing BCR-ABL is responsive to BMS354825 (a dual Src/BCR-ABL kinase inhibitor) and AMN107 (a highly selective BCR-ABL kinase inhibitor) [22,24], we checked the response of MKN74 cells to both inhibitors. Cell viability was significantly decreased in BMS354825-treated (0.01–1.0 μM) MKN74 cells, compared with cells treated with the solvent only, while it was not significantly decreased in AMN107-treated cells (Figure 4A). When the status of CRKL phosphorylation was examined in the MKN74 cells using western blot analysis with an anti-phospho CRKL antibody, CRKL phosphorylation was found to be inhibited more effectively by BMS354825 than by AMN107 (Figure 4B). These results suggested that BMS354825 has the potential to suppress the viability of MKN74 cells expressing CRKL, likely via the inhibition of CRKL phosphorylation.

To further characterize the role of CRKL in the BMS354825-induced suppression of MKN74 cell viability, we examined the effect of BMS354825 on gastric cancer cells without *CRKL* amplification. Since the AGS gastric cancer cell line had lower CRKL mRNA and CRKL protein expression levels than MKN74 cells (Figures 4C and D) and had a normal *CRKL* genomic copy number (Figure 4E), these cells were treated with BMS354825. Unexpectedly, the viability of the BMS354825-treated (0.1–1.0 μM) AGS cells decreased significantly (Figure 4F). Moreover, although the IC_50_ value (inhibitory concentration producing a 50% response) for BMS354825 was slightly higher in AGS cells than in MKN74 cells, the values were not much different between AGS and MKN74 cells (data not shown). These results suggest that BMS354825 has the potential to suppress the viability of AGS cells, likely via a CRKL-independent pathway.

### Decrease in the viability/proliferation of CRKL-expressing MKN74 cells treated with a CRKL-targeting peptide

We then planned to use a more specific inhibitor of CRKL and examined the response of MKN74 and AGS cells to a CRKL-targeting peptide [26]. Cell viability decreased significantly in MKN74 cells treated with the CRKL-targeting peptide (6.25–25 μM), compared with DMSO (solvent)-treated cells, but a similar decrease was not found in AGS gastric cancer cells without *CRKL* amplification (Figure 4G). When cell proliferation was compared after treatment with 6.25 μM of the CRKL-targeting peptide, the cell proliferation was significantly suppressed in MKN74 cells treated with the peptide, compared with DMSO-treated MKN74 cells, but no inhibition of cell proliferation was seen in the AGS cells (Figure 4H). Control peptide had no effect on the gastric cancer cell proliferation. These results suggested that the CRKL-targeting peptide has the potential to suppress the viability/proliferation of gastric cells exhibiting *CRKL* amplification, but not of gastric cells that do not exhibit *CRKL* amplification.

## Discussion

Through a genome-wide SNP microarray analysis performed in this study, the *CRKL* gene was identified as a highly amplified gene in gastric cancer. An increase in the copy number was confirmed in MKN74 gastric cancer cells with *CRKL* amplification using a FISH analysis, and a high CRKL expression level was also observed in these cells. The ability of CRKL to upregulate cell proliferation was shown in MKN74 cells by comparing the cell proliferation rate between CRKL siRNA-transfected cells and negative control siRNA-transfected cells. CRKL protein was overexpressed in 24.4% of the primary gastric cancers, and its level in the gastric cancer was associated with the gender and histopathology. *CRKL* amplification was more frequently found in primary gastric cancers with high CRKL protein expression levels than in those with low CRKL expression levels. Finally, we showed that MKN74 cells with *CRKL* amplification were responsive to the kinase inhibitor BMS354825, likely via the inhibition of CRKL phosphorylation, and a CRKL-targeting peptide. Our current findings suggest that CRKL has an important role in the development of a subset of gastric cancers and has the potential to be a molecular therapy target for gastric cancer.

CRKL is an adaptor cell signaling protein that contains an SH2 domain and two tandem SH3 domains, both of which mediate protein-protein interactions [27,28,30]. CRKL is well known as a surrogate substrate of BCR-ABL kinase in chronic myeloid leukemia and acute lymphoblastic leukemia [11,27,28], and intensive studies of CRKL in Philadelphia chromosome-positive leukemia have been performed. However, only one paper by Kim *et al.*[31] has reported the CRKL status in gastric cancer. They revealed that the expression of CRKL mRNA in a cancer cell line was stimulated by proteins released by *Helicobacter pylori*, although the underlying mechanism was not resolved and the *CRKL* genomic copy number was not analyzed. Our genome-wide SNP microarray analysis successfully revealed, for the first time, that the *CRKL* gene is highly amplified in a subset of gastric cancers. We also showed that the CRKL protein can upregulate cell proliferation using the RNA-interference-mediated knockdown of CRKL in a gastric cancer cell line with *CRKL* amplification. Thus, CRKL overexpression arising from genomic amplification likely contributes to the aggressiveness of gastric cancer.

Recent progress in the development of molecular cancer therapy has revealed new molecular-targeting drugs, such as EGFR-targeting drug ZD1839 (Iressa) and HER2-targeting anti-HER2 monoclonal antibody trastuzumab (Herceptin), to be potent therapies for specific cancers [32-34]. In this study, BMS354825, a dual inhibitor for Src and BCR-ABL kinases, but not AMN107, a BCR-ABL specific inhibitor, showed an inhibitory effect on the survival of MKN74 cells with *CRKL* amplification. A decrease in CRKL phosphorylation through the inhibition of a currently unknown Src kinase seems to be one of the main mechanisms of BMS354825-mediated cytotoxicity in MKN74 cells. BMS354825 is currently being studied clinically in colorectal cancer, prostate cancer, breast cancer, lung cancer, and Philadelphia chromosome-positive leukemia [22,23,35]. Our results suggest that the CRKL protein may be a target of BMS354825-mediated therapy for a subset of gastric cancers. In our analyses, BMS354825 suppressed the viability of AGS cells without *CRKL* amplification as well as the viability of MKN74 cells with *CRKL* amplification, suggesting that a CRKL-independent pathway, which has been previously implicated [36], may also be involved in the BMS354825-mediated cytotoxicity seen in gastric cancers. We also presented the usefulness of a CRKL-targeting peptide for suppressing the proliferation of MKN74 cells with *CRKL* amplification. Our results should contribute to the establishment of CRKL-targeting therapy for a subset of gastric cancers in the future.

In the present study, a genome-wide, high-resolution SNP microarray analysis was successfully performed and five highly amplified chromosome regions containing 22 genes were identified in gastric cancers, as listed in Table 1. Although the *ERBB2* gene, a well-known oncogene that is often amplified in gastric cancer [4], was included in this list, the roles of the most of the genes in the Table have not been studied in gastric cancer. Further investigation of these roles is needed in the future.

## Conclusion

We conclude that CRKL protein is overexpressed in a subset of gastric cancers and is associated with *CRKL* amplification in gastric cancer. Furthermore, we conclude that CRKL protein has the ability to regulate gastric cell proliferation and has the potential to serve as a molecular therapy target for gastric cancer.

## Abbreviations

DAPI: 4',6-diamidino-2-phenylindole; DMSO: Dimethyl sulfoxide; FISH: Fluorescence in situ hybridization; QRT-PCR: Quantitative reverse-transcription-polymerase chain reaction; SNP: Single nucleotide polymorphism; siRNA: Small interfering RNA; TMA: Tissue microarray; SH2/SH3: Src homology 2 and 3.

## Competing interests

The authors declare that they have no competing interests.

## Authors’ contributions

HN performed the experiments and wrote the paper draft. KS and SN interpreted the data and revised the paper. HT, HI, MS, KN, MG, SN, and HY performed a part of the experiments. MM and HK provided tissue samples. SN performed a part of the experiments and was involved in the experimental design. HS conceived the research, designed the experiment, and revised the paper. All authors have read and approved the manuscript.

## References

[B1] Crew KD Neugut AI Epidemiology of gastric cancer World J Gastroenterol 2006 12 354 362 16489633 10.3748/wjg.v12.i3.354 PMC4066052

[B2] Hohenberger P Gretschel S Gastric cancer Lancet 2003 362 305 315 10.1016/S0140-6736(03)13975-X 12892963

[B3] Shinmura K Kohno T Takahashi M Sasaki A Ochiai A Guilford P Hunter A Reeve AE Sugimura H Yamaguchi N Yokota J Familial gastric cancer: clinicopathological characteristics, RER phenotype and germline p53 and E-cadherin mutations Carcinogenesis 1999 20 1127 1131 10.1093/carcin/20.6.1127 10357799

[B4] Tahara E Genetic pathways of two types of gastric cancer IARC Sci Publ 2004 157 327 349 15055305

[B5] Yang S Jeung HC Jeong HJ Choi YH Kim JE Jung JJ Rha SY Yang WI Chung HC Identification of genes with correlated patterns of variations in DNA copy number and gene expression level in gastric cancer Genomics 2007 89 451 459 10.1016/j.ygeno.2006.12.001 17229543

[B6] Sakamoto H Yoshimura K Saeki N Katai H Shimoda T Matsuno Y Saito D Sugimura H Tanioka F Kato S Matsukura N Matsuda N Nakamura T Hyodo I Nishina T Yasui W Hirose H Hayashi M Toshiro E Ohnami S Sekine A Sato Y Totsuka H Ando M Takemura R Takahashi Y Ohdaira M Aoki K Honmyo I Chiku S Aoyagi K Sasaki H Ohnami S Yanagihara K Yoon KA Kook MC Lee YS Park SR Kim CG Choi IJ Yoshida T Nakamura Y Hirohashi S Study Group of Millennium Genome Project for Cancer Genetic variation in PSCA is associated with susceptibility to diffuse-type gastric cancer Nat Genet 2008 40 730 740 10.1038/ng.152 18488030

[B7] Calcagno DQ Leal MF Assumpcao PP Smith MA Burbano RR MYC and gastric adenocarcinoma carcinogenesis World J Gastroenterol 2008 14 5962 5968 10.3748/wjg.14.5962 18932273 PMC2760197

[B8] Shin VY Jin H Ng EK Cheng AS Chong WW Wong CY Leung WK Sung JJ Chu KM NF-κB targets miR-16 and miR-21 in gastric cancer: involvement of prostaglandin E receptors Carcinogenesis 2011 32 240 245 10.1093/carcin/bgq240 21081469

[B9] Mita H Toyota M Aoki F Akashi H Maruyama R Sasaki Y Suzuki H Idogawa M Kashima L Yanagihara K Fujita M Hosokawa M Kusano M Sabau SV Tatsumi H Imai K Shinomura Y Tokino T A novel method, digital genome scanning detects KRAS gene amplification in gastric cancers: involvement of overexpressed wild-type KRAS in downstream signaling and cancer cell growth BMC Cancer 2009 9 198 10.1186/1471-2407-9-198 19545448 PMC2717977

[B10] Feller SM Crk family adaptors-signalling complex formation and biological roles Oncogene 2001 20 6348 6371 10.1038/sj.onc.1204779 11607838

[B11] Birge RB Kalodimos C Inagaki F Tanaka S Crk and CrkL adaptor proteins: networks for physiological and pathological signaling Cell Commun Signal 2009 7 13 10.1186/1478-811X-7-13 19426560 PMC2689226

[B12] Yamamoto G Nannya Y Kato M Sanada M Levine RL Kawamata N Hangaishi A Kurokawa M Chiba S Gilliland DG Koeffler HP Ogawa S Highly sensitive method for genomewide detection of allelic composition in nonpaired, primary tumor specimens by use of affymetrix single-nucleotidepolymorphism genotyping microarrays Am J Hum Genet 2007 81 114 126 10.1086/518809 17564968 PMC1950910

[B13] Ogawa S Nanya Y Yamamoto G Genome-wide copy number analysis on GeneChip platform using copy number analyzer for affymetrix GeneChip 2.0 software Methods Mol Biol 2007 396 185 206 10.1007/978-1-59745-515-2_13 18025694

[B14] Shinmura K Goto M Suzuki M Tao H Yamada H Igarashi H Matsuura S Maeda M Konno H Matsuda T Sugimura H Reduced expression of MUTYH with suppressive activity against mutations caused by 8-hydroxyguanine is a novel predictor of a poor prognosis in human gastric cancer J Pathol 2011 225 414 423 10.1002/path.2953 21826668

[B15] Shinmura K Iwaizumi M Igarashi H Nagura K Yamada H Suzuki M Fukasawa K Sugimura H Induction of centrosome amplification and chromosome instability inp53-deficient lung cancer cells exposed to benzo[a]pyrene diol epoxide (B[a]PDE) J Pathol 2008 216 365 374 10.1002/path.2422 18788085

[B16] Sugimura H Mori H Nagura K Kiyose S Tao H Isozaki M Igarashi H Shinmura K Hasegawa A Kitayama Y Tanioka F Fluorescence in situ hybridization analysis with a tissue microarray: ‘FISH and chips’ analysis of pathology archives Pathol Int 2010 60 543 550 10.1111/j.1440-1827.2010.02561.x 20618731

[B17] Sugimura H Detection of chromosome changes in pathology archives: an application of microwave-assisted fluorescence in situ hybridization to human carcinogenesis studies Carcinogenesis 2008 29 681 687 18283042 10.1093/carcin/bgn046

[B18] Iwaizumi M Shinmura K Mori H Yamada H Suzuki M Kitayama Y Igarashi H Nakamura T Suzuki H Watanabe Y Hishida A Ikuma M Sugimura H Human Sgo1 downregulation leads to chromosomal instability in colorectal cancer Gut 2009 58 249 260 10.1136/gut.2008.149468 18635744

[B19] Suzuki M Nagura K Igarashi H Tao H Midorikawa Y Kitayama Y Sugimura H Copy number estimation algorithms and fluorescence in situ hybridization to describe copy number alterations in human tumors Pathol Int 2009 59 218 228 10.1111/j.1440-1827.2009.02354.x 19351364

[B20] Nakamura S Hirano I Okinaka K Takemura T Yokota D Ono T Shigeno K Shibata K Fujisawa S Ohnishi K The FOXM1 transcriptional factor promotes the proliferation of leukemia cells through modulation of cell cycle progression in acute myeloid leukemia Carcinogenesis 2010 31 2012 2021 10.1093/carcin/bgq185 20823107

[B21] Goto M Shinmura K Nakabeppu Y Tao H Yamada H Tsuneyoshi T Sugimura H Adenine DNA glycosylase activity of 14 human MutY homolog (MUTYH) variant proteins found in patients with colorectal polyposis and cancer Hum Mutat 2010 31 E1861 E1874 10.1002/humu.21363 20848659 PMC3051265

[B22] Rosti G Castagnetti F Gugliotta G Palandri F Martinelli G Baccarani M Dasatinib and nilotinib in imatinib-resistant Philadelphia-positive chronic myelogenous leukemia: a ‘head-to-head comparison’ Leuk Lymphoma 2010 51 583 591 10.3109/10428191003637282 20302388

[B23] Borriello A Caldarelli I Bencivenga D Cucciolla V Oliva A Usala E Danise P Ronzoni L Perrotta S Della Ragione F p57Kip2 is a downstream effector of BCR-ABL kinase inhibitors in chronic myelogenous leukemia cells Carcinogenesis 2011 32 10 18 10.1093/carcin/bgq211 20952511

[B24] Weisberg E Manley PW Breitenstein W Brüggen J Cowan-Jacob SW Ray A Huntly B Fabbro D Fendrich G Hall-Meyers E Kung AL Mestan J Daley GQ Callahan L Catley L Cavazza C Azam M Neuberg D Wright RD Gilliland DG Griffin JD Characterization of AMN107, a selective inhibitor of native and mutant Bcr-Abl Cancer Cell 2005 7 129 141 10.1016/j.ccr.2005.01.007 15710326

[B25] Sugimoto Y Nakamura S Okinaka K Hirano I Ono T Shigeno K Shinjo K Ohnishi K HOXA10 expression induced by Abl kinase inhibitors enhanced apoptosis through PI3K pathway in CML cells Leuk Res 2008 32 962 971 10.1016/j.leukres.2007.11.034 18190961

[B26] Kardinal C Konkol B Schulz A Posern G Lin H Adermann K Eulitz M Estrov Z Talpaz M Arlinghaus RB Feller SM Cell-penetrating SH3 domain blocker peptides inhibit proliferation of primary blast cells from CML patients FASEB J 2000 14 1529 1538 10.1096/fj.14.11.1529 10928987

[B27] Sattler M Salgia R Role of the adapter protein CRKL in signal transduction of normal hematopoietic and BCR/ABL-transformed cells Leukemia 1998 12 637 644 10.1038/sj.leu.2401010 9593259

[B28] ten Hoeve J Arlinghaus RB Guo JQ Heisterkamp N Groffen J Tyrosine phosphorylation of CRKL in Philadelphia + leukemia Blood 1994 84 1731 1736 7521685

[B29] Nakamura T Komiya M Sone K Hirose E Gotoh N Morii H Ohta Y Mori N Grit, a GTPase-activating protein for the Rho family, regulates neurite extension through association with the TrkA receptor and N-Shc and CrkL/Crk adapter molecules Mol Cell Biol 2002 22 8721 8734 10.1128/MCB.22.24.8721-8734.2002 12446789 PMC139861

[B30] ten Hoeve J Morris C Heisterkamp N Groffen J Isolation and chromosomal localization of CRKL, a human crk-like gene Oncogene 1993 8 2469 2474 8361759

[B31] Kim N Park WY Kim JM Park JH Kim JS Jung HC Song IS Gene expression of AGS cells stimulated with released proteins by Helicobacter pylori J Gastroenterol Hepatol 2008 23 643 651 10.1111/j.1440-1746.2007.05241.x 18070016

[B32] Ko JC Ciou SC Cheng CM Wang LH Hong JH Jheng MY Ling ST Lin YW Involvement of Rad51 in cytotoxicity induced by epidermal growth factor receptor inhibitor (gefitinib, IressaR) and chemotherapeutic agents in human lung cancer cells Carcinogenesis 2008 29 1448 1458 10.1093/carcin/bgn130 18544565

[B33] Holden J Garrett Z Stevens A NICE guidance on trastuzumab for the treatment of HER2-positive metastatic gastric cancer Lancet Oncol 2011 12 16 17 10.1016/S1470-2045(10)70276-X 21249723

[B34] Norell H Poschke I Charo J Wei WZ Erskine C Piechocki MP Knutson KL Bergh J Lidbrink E Kiessling R Vaccination with a plasmid DNA encoding HER-2/neu together with low doses of GM-CSF and IL-2 in patients with metastatic breast carcinoma: a pilot clinical trial J Transl Med 2010 8 53 10.1186/1479-5876-8-53 20529245 PMC2903523

[B35] Araujo J Logothetis C Dasatinib: a potent SRC inhibitor in clinical development for the treatment of solid tumors Cancer Treat Rev 2010 36 492 500 10.1016/j.ctrv.2010.02.015 20226597 PMC3940067

[B36] Kantarjian HM Cortes J La Rosée P Hochhaus A Optimizing therapy for patients with chronic myelogenous leukemia in chronic phase Cancer 2010 116 1419 1430 10.1002/cncr.24928 20120030

